# Disinformation and Conspiracy Theories in the Age of COVID-19

**DOI:** 10.3389/fsoc.2020.560681

**Published:** 2020-11-12

**Authors:** Pedro Silveira Pereira, Antonio da Silva Silveira, Antonio Pereira

**Affiliations:** ^1^Independent Researcher, Belém, Brazil; ^2^Department of Electrical and Biomedical Engineering, Institute of Technology, Federal University of Pará (UFPA), Belém, Brazil

**Keywords:** COVID-19, coronavirus, disinformation, misinformation, ideology, conspiracy theory, cognitive bias

Since the 11th of March 2020, the 2019 coronavirus disease (COVID-19) has been declared a global pandemic by the (World Health Organization, 2020). The disease is caused by the SARS-CoV-2 and was first officially reported in Wuhan, China, in December 2019 (Zhu et al., 2020). Since then, COVID-19 has spread globally with millions of laboratory-confirmed cases and hundreds of thousands of deaths (Relief Web, 2020). So far, there is no specific treatment for the disease and many research teams are currently working on a vaccine that, optimistically, will only be available to the public in 2021. Meanwhile, the recommendation from health authorities is to adopt nonpharmaceutical interventions such as travel restrictions, school closures, social distancing, washing hands, and wearing face masks. Though these emergency measures are certainly inconvenient, social distancing has been proven historically effective in reducing and delaying infection rates and mortality on previous influenza pandemics (1918 and 2009) (Ahmed et al., 2018) while face masks minimize the risk of spreading viral particles through respiratory droplets (Leung et al., 2020). In short, the greater part of the success of mitigation strategies depends on individual responsibilities for implementing the recommended personal-level actions.

Unfortunately, however, social distancing guidelines against COVID-19 have become a political hot topic and compliance has roughly been defined along ideological lines: conservatives are less probable to adhere to them than liberals (Rothgerber et al., 2020). To complicate matters, there has been a flood of conspiracy theories and false news about COVID-19. For instance, the conspiracy theory that the coronavirus is a laboratory-engineered bioweapon created by the Chinese started in January 2020 and was spread, bot-like, in Twitter by mostly right-wing and conservative profiles (Graham et al., 2020). While conspiracy theories are not the preserve of the ideological left or right, they are more common at ideological extremes and certainly strongest at the extreme right (Sutton and Douglas, 2020). The appeal of conspiracy theories is that they often serve as a “radicalizing multiplier” (Bartlett and Miller, 2010) for fringe groups while offering an easy explanation for complex issues (Marchlewska et al., 2018), thus satisfying people's need for cognitive closure (Kruglanski and Fishman, 2009). However, as seen with “the stab in the back” myth in Germany after the end of WWI, for instance, the unchallenged dissemination of conspiracy theories and false news can posit a great risk to democracy (Ardèvol-Abreu et al., 2020).

Aided by the existence of modern information networks powered by the internet, coordinated disinformation campaigns disseminating conspiracy theories, false news, and health hoaxes, are more common than ever. Conspiracy theories usually have a system-justifying function of supporting the status quo by redirecting the public attention toward imaginary perils and distracting from genuine threats (Eco, 2014; Jolley et al., 2018). Health hoaxes and false news also sidetrack demands for adequate and science-backed solutions to fight the pandemic and its consequences, such as investment on vaccine development, adequate hospital infrastructure (ventilators, ICU units, etc.), and financial relief programs. Some conservative political leaders have regularly stressed the link between the adoption of social distancing guidelines with negative effects on the economy, even though there is evidence from the 1918 influenza pandemic that US cities that moved more aggressively to limit interactions among the public fared much better economically afterward than cities which were laxer (Correia et al., 2020). To justify the end of lockdowns, some have also promoted the use of unproven therapeutic methods, such as Chloroquine (CQ)/hydroxychloroquine (HCQ), to treat COVID-19 (Guzman-Prado, 2020).

CQ was proposed in the 1930s as a drug to treat malaria (Peters, 1971), which is still the deadliest infectious disease in the world. HCQ was later introduced as a less toxic version of the drug and was approved to treat autoimmune diseases (Ben-Zvi et al., 2012). CQ and HCQ garnered worldwide attention as promising candidates to treat COVID-19 in early February 2020 after the publication of reports showing *in vitro* activity of CQ against severe acute respiratory syndrome coronavirus 2 (SARS-CoV-2) (Wang et al., 2020). Subsequently, several randomized controlled clinical trials were initiated but none was able to prove its efficacy against COVID-19 (Recovery, 2020) and some were halted due to the possibility of harmful side effects. Meanwhile, beginning on 19 Mar 2020, President Donald Trump promoted the use of CQ/HCQ as a game-changer against COVID-19. Other conservative leaders around the world followed suit and began promoting the use of the drugs in their own countries as well. In the USA, the hype with chloroquine was short-lived due to counter-recommendations from the Food and Drug Administration (FDA) (US FDA, 2020), but in other countries, such as Brazil, it never went away due to official support for its use (See Figure 1). As shown in Figure 1 comparing the US with two other countries in the Americas (Argentina and Brazil), Google searches for CQ/HCQ spiked in response to President Trump's press meeting on 19 Mar 2020 not only in the US but in both Argentina and Brazil. Afterward, the number of searches subsided, except in Brazil, where government officials have promoted CQ/HCQ as a valid therapy against COVID-19 even though there is no availability of clinical trial data regarding its safety and efficacy (Chowdhury et al., 2020).

**Figure 1:**
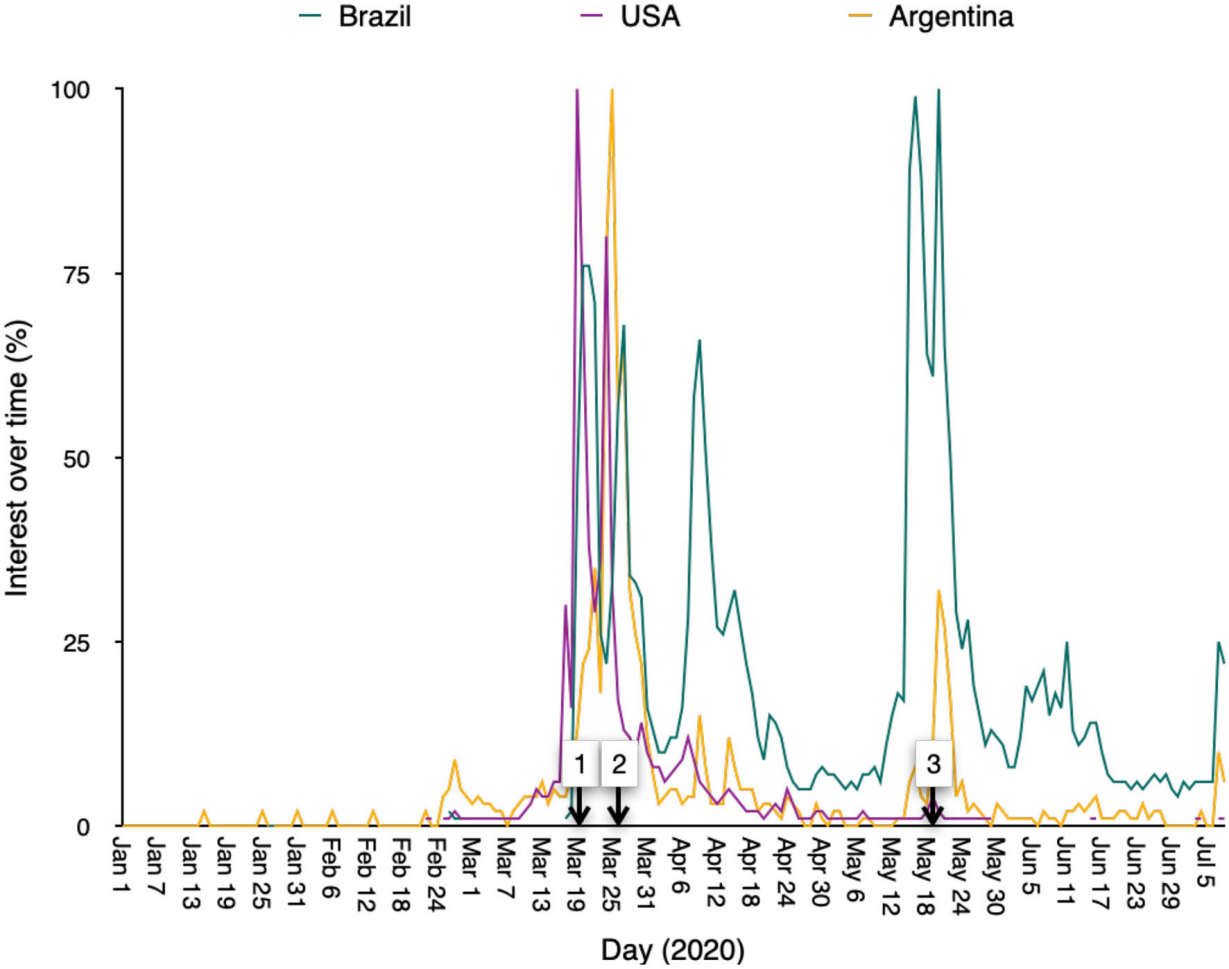
The arrow marks the day that U.S. President Donald Trump held a news briefing saying the government would make the drug available “almost immediately” to treat COVID-19 (03/19/20). On 03/25/20, Brazil's President posted about the benefits of chloroquine. On 05/20/20 Brazil's Ministry of Health issued a protocol to treat COVID-19 patients with chloroquine.

Even though most people obtain the news from conventional media outlets such as television and newspapers, not from social network applications or false news (Allen et al., 2020), heads of government have a bully pulpit through which they can reach a wider audience via traditional media coverage. In our polarized political times, their message is also propagated by both supporters and non-supporters in social media. Besides, the filtering technologies currently used by social media platforms facilitate the formation of psychosocial bubbles that limit the diversity of social contacts and feed the so-called digital “echo chambers” (Kaakinen et al., 2020).

The main assumption of the social identity approach (SIA) is that each person not only has a distinct personal identity but also social identities that connect them to other people (Brown, 2000). According to the SIA, group memberships are important parts of a person's self-concept and shapes a person's experience of the world (Hornsey, 2008). For instance, it is known that personal ideology influences people's opinions on climate change policy (McCright and Dunlap, 2011; Fielding et al., 2020) and influence their decision to share false and misleading content, even though they generally wish to avoid spreading misinformation and are often able to tell truth from falsehood (Pennycook et al., 2019). Thus, by stressing the notion of “us” against “them,” the promoters of conspiracy theories and false news can vastly increase the chance of their message being spread.

The backlash against science-based methods to fight infectious diseases is not new. For instance, anti-vaccination movements were common in the 19th century in England, the US, and Brazil (Figure 2) (Jolley and Douglas, 2014). What's new is the social environment for the propagation of contrarian views. For most online extremists, the content of the message does not matter as much as its potential to be used as a bait to amplify the visibility of a conspiracy theory to the wider public when mainstream media and prominent social media actors engage with the conspiracy theory, even critically. Even official denials and corrections can be exploited by conspiracy theorists to claim that authorities are covering up “the real truth” (Graham et al., 2020). Conspiracy theories promoted by the anti-vaccination movement have been widely circulated in social media in recent years and could even hamper the efforts to reach a larger share of the population with an eventual COVID-19 vaccine (Megget, 2020).

**Figure 2:**
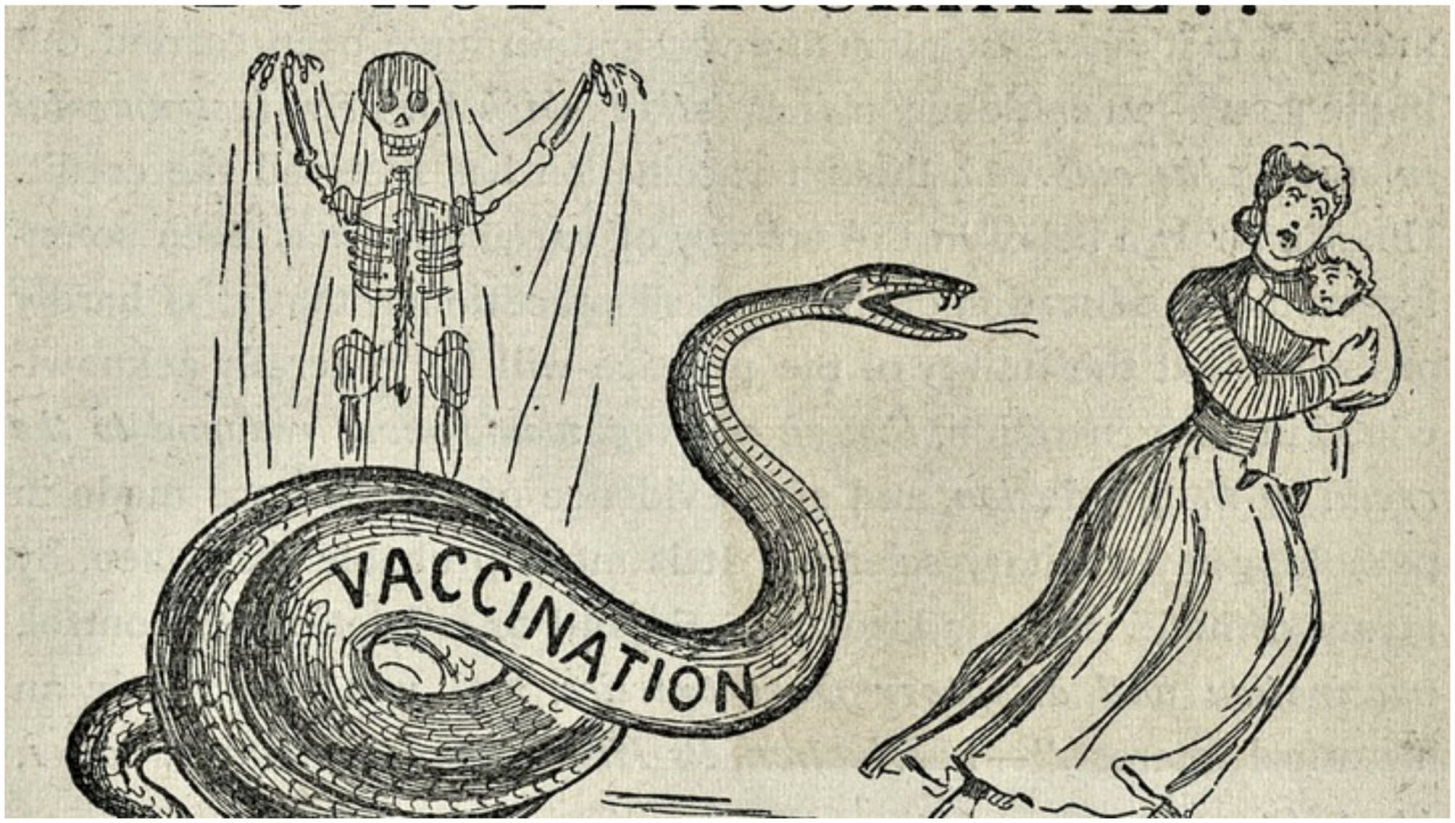
A illustration from an 1894 anti-vaccination publication (The Historical Medical Library of the College of Physicians of Philadelphia).

A recent study showed that misinformation about COVID-19 on Facebook is available in several languages and much of this content remains active in the platform without a warning or label, giving ample time for it to go viral (Avaaz, 2020b). In a joint statement, Facebook, Google, LinkedIn, Microsoft, Reddit, Twitter, and YouTube have vowed to work against misinformation in their platforms (Shu and Shieber, 2020). However, some observers agree that more has to be done by these companies, including correct the record on health misinformation by individually sending warnings to recipients of false news, ban repeated offenders, and change their algorithms to prevent their posts of appearing systematically on feeds (Avaaz, 2020b). Facebook's algorithm, for instance, rewards and encourages user's engagement with content that provokes strong emotions, which is usually how false information is packaged: as something novel and sensational (Avaaz, 2020a).

Thus, there is a strong need for a vast campaign led by respected institutions and individuals to advise the public to be cautious with dubious claims of effective therapies for COVID-19 and other infectious diseases. A recent proposal is to implement a suite of interventions based on accuracy nudges to make people think about the accuracy of the information they want to share in social platforms (Pennycook et al., 2020). Also, factually inaccurate information disseminated in social media should be promptly labeled and/or removed by social media outlets. Unfortunately, only tagging such stories as inaccurate, as done by Twitter, for instance, does not seem to be an effective solution to this problem (Pennycook et al., 2018). However, to preserve fundamental free-speech rights, moderation decisions should be carried with the utmost transparency by non-governmental oversight boards selected to represent society's diversity. Moderating decisions should be explained in the most user-friendly way to the public. Although there is a strong debate on the effectiveness of corrective measures (Jerit and Zhao, 2020), recent research shows that repeated exposure to correct information contributes to repair the damage of viral misinformation spread in the realm of social media (Carnahan et al., 2020). These measures are a small but necessary step in building a confidence society, where mistrust and pessimism do not further corrode the social tissue (Collectif, 2016).

Infectious diseases have always been an existential threat to mankind (Shaw-Taylor, 2020). Before the emergency of antibiotics or vaccines, i.e., for most of human history, the unexpected introduction of infectious agents could mean the decimation of some immunologically naïve groups. Besides the physiological immune system, we evolved behavioral immune responses that protect us against pathogen threats and infectious hazards in a more proactive way (Schaller, 2011). Those responses, however, operate mainly subconsciously (Mercier, 2020), and similar to other evolved threat management systems, behavioral immune responses are characterized by contextual sensitivity and biases that aid adaptive responding (Haselton et al., 2015; Ackerman et al., 2018). Though people are usually wary of other people's opinions or advice (Trouche et al., 2018), they are susceptible to repetition, i.e., repeated statements tend to be rated as more likely to be true (Trouche et al., 2018), the so-called “illusory truth effect” (Hasher et al., 1977; Pennycook et al., 2018). During times of elevated stress, such as the ongoing pandemic, our faulty decision-making heuristics are more susceptible to be targeted by groups trying to control the public narrative to their benefit (Starcke and Brand, 2012). Though this procedure is not new, the danger to public health demands a prompt response from society. Words have consequences, and they have been used carelessly in the current pandemic by elected officials, contributing to confuse the public and discredit scientific expertise in the fight against SARS-CoV-2.

## Author Contributions

PP, AS, and AP wrote the manuscript. All authors contributed to the article and approved the submitted version.

## Conflict of Interest

The authors declare that the research was conducted in the absence of any commercial or financial relationships that could be construed as a potential conflict of interest.
